# Current Trends in the Treatment of Cervical Pregnancy: A Narrative Review

**DOI:** 10.3390/medicina61112072

**Published:** 2025-11-20

**Authors:** Nikoleta Stoyanova, Angel Yordanov, Nikola Popovski

**Affiliations:** 1 Department of Obstetrics and Gynaecology, Medical University Pleven, 5800 Pleven, Bulgaria; niikoletastoyanova@gmail.com (N.S.); nikola.popovski@mu-pleven.bg (N.P.); 2 Department of Gynaecological Oncology, Medical University Pleven, 5800 Pleven, Bulgaria

**Keywords:** cervical pregnancy, ectopic pregnancy, management, treatment

## Abstract

*Background and Objective*: Cervical pregnancy, as a rare type of ectopic pregnancy, can lead to life-threatening complications. Early diagnosis is crucial for optimal management and fertility-sparing strategies. However, despite advances in imaging and early detection, standardized guidelines for management are still lacking. *Materials and Methods*: This narrative review is based on the collection of case reports of CEP, published in PubMed and Google Scholar in the period 1984–2025. We also included heterotopic cervical pregnancy as it poses even greater challenge to the clinicians. *Results*: Twenty-two articles reporting a total of twenty-four case reports specifically focus on different management techniques and their corresponding success rates. Currently, there is no consensus regarding the optimal management of this type of ectopic pregnancy, and therapeutic decisions are largely influenced by the clinical presentation, available resources, and the expertise of the treating clinicians and institutions. *Conclusions*: Conservative approaches should be prioritized as first-line therapy in hemodynamically stable patients where fertility preservation is desired. Minimally invasive procedures may be considered as second-line options following failure of conservative management. Hysterectomy remains the last resort for patients with refractory disease or life-threatening hemorrhage. The proposed algorithm provides an expert-based framework for the management of cervical ectopic pregnancy, emphasizing hemodynamic stability, β-hCG levels, and fertility preservation as key determinants of treatment selection.

## 1. Introduction

Cervical pregnancy (CEP) represents a rare but potentially life-threatening form of non-tubal ectopic pregnancy, characterized by implantation and development of the embryo inside the endocervical canal [1]. It accounts for less than 1% of all ectopic pregnancies, with an estimated incidence of approximately 1 in 10,000 live births [2]. Owing to its rarity, epidemiology and risk factors remain insufficiently understood, although prior uterine curettage, cesarean delivery, cervical surgical procedures, intrauterine device use and pelvic infections have been suggested [3,4,5]. The association with assisted reproductive technologies (ART) is debated, with recent evidence showing no significant impact of IVF on CEP incidence [2,3]. Diagnosis is usually based on a combination of clinical presentation, transvaginal ultrasound findings, and, in some cases, histopathological confirmation [4]. Although surgical management has long been the standard, growing evidence supports medical or combined conservative approaches in selected cases, achieving favorable outcomes [5]. The rarity of CEP, combined with the absence of standardized diagnostic criteria and the wide variability in management strategies, has led to a literature base dominated by individual case reports and small series. Consequently, there is limited high-quality evidence to guide clinical decision-making. Given these challenges and the evolving trends in both medical and surgical management, a comprehensive review of current treatment strategies is warranted to consolidate existing knowledge, highlight potential best practices, and identify areas in need of further research.

## 2. Methodology

This narrative review summarizes and critically appraises the current knowledge on cervical ectopic pregnancy, focusing on risk factors, diagnostic criteria, and management strategies. Although not conducted as a systematic review, the search and selection process followed a structured and transparent approach to ensure comprehensive coverage of the available literature.

### 2.1. Search Strategy

In light of the poorly defined etiology, absence of standardized diagnostic guidelines and unified therapeutic approach, we performed literature searches of PubMed and Google Scholar for studies published between 1 January 1984 and 1 September 2025. The primary search string used in PubMed was: (“cervical ectopic pregnancy” OR “cervical pregnancy” OR “ectopic cervical pregnancy”) AND (treatment OR management OR therapy OR methotrexate OR surgery). An analogous strategy was applied to Google Scholar. Reference lists of included articles and relevant reviews were hand-searched to identify additional reports.

### 2.2. Eligibility Criteria

Inclusion criteria were: (1) case reports and case series of patients diagnosed with cervical ectopic pregnancy of any gestational age; (2) diagnosis confirmed by ultrasound or other accepted diagnostic modalities; (3) patients who received medical and/or surgical treatment; (4) full-text articles available in English; and (5) publications between 1984 and 2025. Exclusion criteria were: (1) non-full-text reports; (2) publications in languages other than English; (3) reviews, editorials, conference abstracts without patient-level data; (4) ectopic pregnancies at non-cervical sites (e.g., tubal, ovarian, abdominal); and (5) duplicate reports of the same patient.

### 2.3. Study Selection and Data Extraction

**Figure 1:**
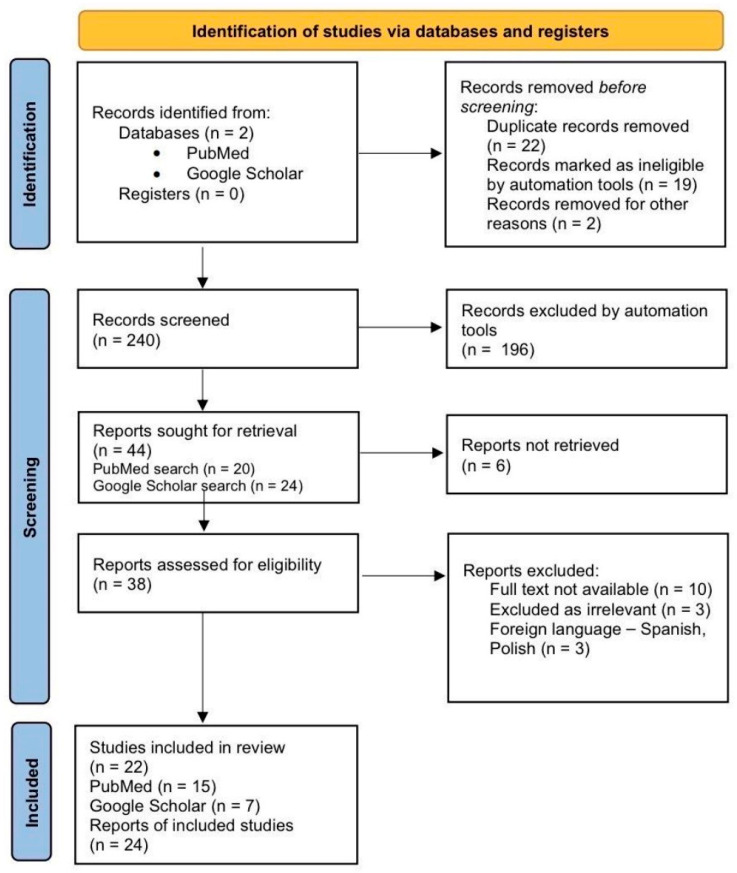
Flowchart illustrates the literature search and selection process for the case reports analyzed in the review and discussion.

All records retrieved from the databases were exported to a reference manager, and duplicate entries were removed. The study selection process is summarized in the flowchart on Figure 1. A total of 283 records were initially identified—163 from PubMed and 120 from Google Scholar. After removal of 22 duplicate records and the exclusion of 196 records by automation tools (based on title and abstract relevance), 44 records remained for manual screening. Of these, 38 full-text articles were assessed for eligibility. Reasons for exclusion at the full-text review stage included: not a cervical ectopic pregnancy (*n* = 3), no patient-level data (*n* = 3), non-English language (*n* = 3), and lack of full-text availability (*n* = 10). Ultimately, 22 articles met the inclusion criteria and were included in the qualitative synthesis, comprising a total of 24 reported cases (15 from PubMed and 7 from Google Scholar). The completeness of reporting for each case and variable is summarized in Supplementary Table S1.

### 2.4. Data Items and Outcomes

From each included report we extracted: maternal age, parity, relevant medical history (including known risk factors), method of conception, method of diagnosis, gestational age at diagnosis, initial β-human chorionic gonadotropin (β-hCG) level, and the type of medical or surgical treatment. For medical management we recorded specific agents and routes of administration; for surgical management we recorded the technique used, intraoperative and postoperative complications, need for additional interventions, and maternal outcomes. Where reports lacked specific data for an item, that study was excluded from analyses of that particular variable and this is indicated in the tables and text.

#### Risk of Bias and Level of Evidence

Given that included studies were case reports and case series (low level of evidence), we did not perform a formal risk-of-bias tool designed for randomized or observational comparative studies. Instead, we assessed completeness of reporting (diagnostic confirmation, hCG values, follow-up duration, and outcome clarity) and performed sensitivity analyses by excluding reports with critically incomplete data to evaluate the robustness of synthesized conclusions.

### 2.5. Data Synthesis

Because of heterogenicity in patient characteristics, diagnostic criteria, interventions, and outcome reporting, we performed a qualitative, comparative synthesis rather than a meta-analysis. Frequencies and descriptive statistics are reported where data were sufficiently complete. Heterogenicity was explored qualitatively by comparing clinical and procedural characteristics across reports. Sensitivity analyses excluded cases with insufficient diagnostic confirmation or missing primary outcome data. Table 1 summarizes the characteristics of the patients reported in the included studies.

## 3. Results

We observed the following conclusions: Of the 24 included cases, reporting completeness varied across studies. Maternal age was available in 22/24 cases, parity in 23/24, gestational age in 22/24, β-hCG levels in 14/24, and treatment outcomes in all 24. The completeness of reporting for each variable is summarized in Supplementary Table S1.

### 3.1. Patient Characteristics

The maternal age ranges between 22 and 45 years. In our cohort, 41% (10 out of 24) of the patients were above the age of 35.

Parity indicates a predominance of women with previous abortions or sterility (e.g., G3P0A2, G7P0A5), suggesting a history of reproductive difficulties.

The most common gestational age at diagnosis is between 5 and 10 weeks of gestation, but diagnosis at later stage (e.g., 12 g.w.) is also described.

### 3.2. Risk Factors

**Figure 2:**
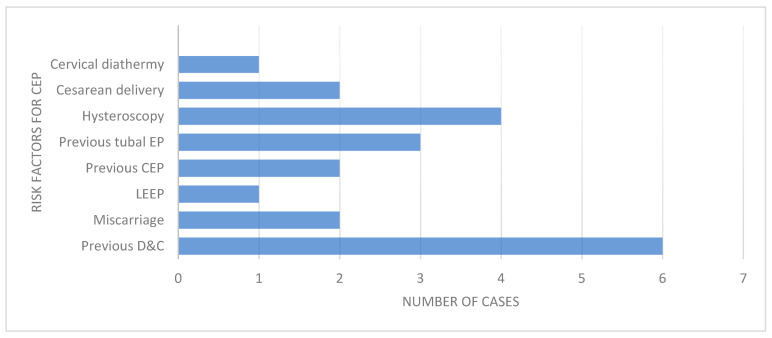
Graph of frequency of risk factors for CEP.

Commonly reported risk factors included a history of uterine curettage (21%), prior ectopic pregnancy or salpingectomy (13%), and infertility managed with assisted reproductive technologies (IVF/ICSI in 30%). Additional risk factors involved cervical interventions such as loop electrosurgical excision procedure (LEEP), cervical diathermy, and hysteroscopy. Figure 2 summarizes the frequency of risk factors. Prior D&C/D&E procedures were among the most common risk factors identified similarly to previous studies on the topic.

Spontaneous conception was the most common mode of conception, accounting for more than half of the reported cases. However, assisted reproductive technologies (ART), particularly in vitro fertilization (IVF) and intracytoplasmic sperm injection (ICSI), were involved in at least six cases. These were often associated with heterotopic cervical pregnancies, especially among women with a history of infertility, tubal pathology, or prior reproductive surgeries such as salpingectomy or hysteroscopic procedures.

### 3.3. Diagnostic Methods

Transvaginal ultrasound (TVUS) served as the primary diagnostic tool and was utilized in all cases with a confirmed diagnosis.

An exception was the case reported by Kouyoumdjian et al., in which no imaging was performed due to the historical context—predating the routine use of ultrasound—and the diagnosis was established retrospectively via histological examination [17]. Notably, heterotopic cervical pregnancies were identified in six cases, predominantly in the context of IVF. These cases underscore the importance of thorough sonographic evaluation, particularly in ART-conceived pregnancies where the risk of multiple implantation sites is elevated.

### 3.4. Treatment Modalities

Conservative management using Methotrexate (MTX) represents one of the most commonly used treatment strategies for cervical pregnancy. MTX was administered via intramuscular injection of MTX 50 mg/m^2^ (most frequently), intra-amniotic instillation or within multi-dose protocols (e.g., on days 1, 3, 5 and 7). Out of a total of 24 cases, MTX was utilized as the initial treatment in 11 cases. In 5 of these, MTX therapy was successful as a stand-alone intervention, with no need for additional procedures. However, in the remaining 6 cases, treatment with MTX alone was insufficient, necessitating further interventions due to persistent embryonic viability or the onset of significant vaginal bleeding. These additional procedures included dilation and curettage (D&C), selective uterine artery branch embolization (SUABE), or uterine artery embolization (UAE).

In severe or refractory cases, surgical management, such as hysterectomy, was necessary, particularly in the context of uterine perforation or life-threatening hemorrhage. Embolization procedures were often followed by ultrasound monitoring to confirm reduced vascularity and treatment success.

Table 2 summarizes treatment outcomes. Methotrexate alone was successful in approximately 45% of cases, while combined or interventional approaches such as embolization achieved higher success rates.

### 3.5. Complications

Complications primarily included severe vaginal bleeding, which was the leading adverse event following curettage or failed conservative treatment. Other complications such as uterine perforation, hysterectomy (as a life-saving measure), and rehospitalization for delayed bleeding or infection were also reported. Severe complications, mainly massive vaginal bleeding, occurred in approximately 17% of all cases, while hysterectomy was required in 12%. These findings highlight that early diagnosis and timely, minimally invasive treatment can substantially reduce morbidity and the need for radical surgery. Table 3 summarizes the frequency of reported complications among the included cases.

### 3.6. Outcomes

In most cases, β-hCG normalizes within a few weeks.

Favorable reproductive outcomes have been achieved in numerous cases, including: successful intrauterine pregnancies after treatment of CEP, preservation of intrauterine pregnancy in heterotopic CEP and delivery at term.

## 4. Discussion

### 4.1. Clinical Significance and Risk Factors

Although CEP accounts for less than 1% of all ectopic pregnancies, it carries a disproportionately high risk of severe hemorrhage, hysterectomy, or maternal death if not promptly diagnosed and managed. The clinical challenge becomes greater in cases of coexisting intrauterine pregnancy, where treatment must balance maternal safety with the potential viability of the intrauterine gestation.

Previous uterine instrumentation, particularly dilation and curettage (D&C), has consistently emerged as the most important risk factor, reported in up to 80% of cases. The association with assisted reproductive technology (ART) also suggests a possible role of altered embryo transfer dynamics. Our findings are consistent with this literature, as most patients in our series had a prior history of D&C.

### 4.2. Diagnosis: Advances and Challenges

Early diagnosis is essential to reduce morbidity. Historically, CEP was often recognized only when hemorrhage occurred, frequently leading to hysterectomy. Today, transvaginal ultrasound, complemented by Doppler, has become the cornerstone of diagnosis. Criteria such as the “sliding sign” help distinguish CEP from miscarriage and allow earlier, fertility-preserving intervention. While MRI has been described, its added value remains limited [28]. The key clinical implication is that timely use of ultrasound has transformed CEP from a life-threatening condition into one that can often be conservatively managed.

Several clinical signs are suggested to help diagnosis of CEP such as (1) painless uterine bleeding after period of amenorrhea, (2) hourglass shape of the uterus due to enlarged cervix equal to or even larger than uterine fundus, (3) pregnancy tissue located within the cervical canal, (4) closed internal cervical os, (5) partially opened external cervical os [29,30].

The first ultrasound description of cervical pregnancy was reported by Raskin in 1978 [31]. Key sonographic criteria are used to diagnose CEP, as summarized in Table 4 (1) enlarged cervix, (2) enlarged uterus, (3) presence of diffuse, irregular echoes within the uterus, (4) absence of intrauterine pregnancy, (5) detection of peritrophoblastic blood flow around the gestational sac using color Doppler, (6) absence of the “sliding sign,” which refers to the inability of the gestational sac to move inside the cervical canal when gentle pressure is applied with a transvaginal ultrasound probe. Jurkovic et al. proposed “sliding sign” as a diagnostic criteria to distinguish CEP from a miscarriage [9,32]. An additional distinguishing feature is the absence of peritrophoblastic flow around non-viable sac passing through the cervix in the cervical stage of a miscarriage [15]. Other sonographic findings that may help differentiate CEP from a cervical miscarriage include the frequent presence of fetal cardiac activity, an ‘hourglass-shaped’ uterus, a regular echogenic decidual reaction or a pseudogestational sac within the endometrium, and a closed internal os, which is often observed in cases of CEP.

### 4.3. Treatment Approaches

Treatment of CEP still remains challenging due to its rarity, which limits clinical experience, the difficulty in early and accurate diagnosis, and the potential for severe hemorrhagic complications requiring multidisciplinary management. The therapeutic goal is to balance maternal safety with fertility preservation whenever possible.

In the past, hysterectomy was the primary treatment modality for CEP due to lack of minimally invasive alternatives and high risk of life-threatening hemorrhage [33]. Nowadays, advances in imaging, allowing early diagnosis and fertility-sparing interventions have enabled more conservative approaches in selected cases.

In Bulgaria there are no guidelines for management of CEP. Based on the literature data Methotrexate is widely considered first-line in selected cases. Both systemic and local administration are used, with local injection offering higher efficacy and fewer systemic side effects, though requiring specialized expertise [1]. Prognostic factors such as serum β-hCG > 10,000 mIU/mL or advanced gestational age reduce MTX success rates [22]. Combination regimens (e.g., MTX with KCl or mifepristone) may improve outcomes but carry variable risks [1,34]. Petousis S. et al. reported successful outcome of application of single dose MTX followed by intra-amniotic KCl injection [35], while Minnini et al. reported a case associated with massive vaginal bleeding three months later, necessitating uterine artery embolization [8]. However, in 2002 Sexton and Sharp reported the first clinical case of a patient with CEP successfully treated with MTX and Mifepristone [1,36].

D&C, historically used, carries a high hemorrhage risk and is safer when combined with adjunctive measures such as Foley balloon tamponade or medical therapy [13,37]. Hysteroscopic approaches have gained attention, showing reduced morbidity compared to D&C in selected cases.

Maglic et al. reported small-caliper hysteroscopy as a successful treatment method comparing outcomes between patients treated with this technique and those who underwent D&C [38]. Their results demonstrate that patients treated with small-caliber hysteroscopy experienced reduced blood loss and shorter hospital stays compared to the D&C group.

Cvetkov D. et al. reported, for the first time, the successful use of the Ho:YAG laser in the treatment of cervical pregnancy [27].

Invasive methods for treatment have also been described. Uterine artery embolization (UAE) and super-selective embolization of the pathological uterine arteries branch (SUABE) are methods of choice in patients with massive vaginal bleeding or after unsuccessful outcome from conservative treatment. Takeda et al. reported a treatment protocol involving bilateral uterine artery embolization followed by intramuscular MTX administration on the first postoperative day [39]. Gun et al. reported a successful outcome following bilateral UAE performed in response to severe vaginal bleeding that occurred after systemic methotrexate administration [15]. However, there is risk of recurrent massive bleeding following UAE, particularly in cases with fetal cardiac activity prior to the procedure, persistent elevated beta-hCG levels and the reappearance of blood flow signals around the gestational sac [40]. These factors highlight the importance of ultrasound confirmation to assess the success of the intervention.

Hysterectomy remains a definitive option when conservative measures fail or fertility preservation is not desired. Alammari R. et al. reported a case of CEP treated successfully via vaginal hysterectomy [41].

More recently, lauromacrogol, a sclerosing foam agent, has been introduced as a novel minimally invasive treatment option for cervical pregnancy. Zhang et al. reported two successful cases of cervical pregnancy managed with lauromacrogol injection combined with an intrauterine visualization system and vacuum aspiration. The treatment demonstrated rapid decline in serum β-hCG levels, minimal intraoperative bleeding, and quick postoperative recovery [42].

Our findings are in line with the conclusions of recent systematic reviews, which emphasize that conservative and fertility-preserving approaches should be considered first-line treatment options for cervical ectopic pregnancy, while minimally invasive methods represent valuable secondary interventions in selected cases [37,43].

Given the heterogeneous findings on different treatment approaches, a critical appraisal of the current evidence and its implications for clinical practice is warranted.

### 4.4. Critical Appraisal, Limitations, and Implications for Practice

#### 4.4.1. Critical Appraisal and Limitations

Given the rarity of cervical pregnancy, the existing literature is largely composed of individual case reports and small case series, which provide a low level of evidence (Level IV–V). As a result, the ability to draw broad, generalizable conclusions or to establish standardized management protocols remains limited. These reports are often heterogeneous regarding diagnostic criteria, treatment strategies, and follow-up, which complicates comparison and synthesis of data. Moreover, publication bias likely favors successful conservative cases, leading to overestimation of treatment efficacy.

Another limitation is the absence of randomized or prospective data comparing different therapeutic approaches. Most cases are managed based on institutional expertise rather than standardized guidelines, resulting in significant variability in treatment selection and outcomes. Furthermore, β-hCG thresholds predicting methotrexate success remain inconsistent across studies.

In addition, incomplete reporting across case reports represents a further limitation. Not all studies provided data for every analyzed parameter, which may introduce selective reporting bias. Although missing data were handled by excluding incomplete cases from specific analyses, this approach may underestimate or overestimate the frequency of certain features (e.g., risk factors or complications). The Supplementary Table S1 was created to increase transparency regarding data completeness.

#### 4.4.2. Implications for Clinical Practice

Despite these limitations, several practical conclusions can be drawn. Early diagnosis via transvaginal ultrasound remains the cornerstone of management and allows fertility-sparing treatment in hemodynamically stable patients. Methotrexate, either systemically or locally administered, is an effective first-line therapy in selected cases with low β-hCG levels and absent fetal cardiac activity. In cases with active bleeding or methotrexate failure, uterine artery embolization or surgical management (D&C, hysteroscopy, or hysterectomy) remain essential options. A multidisciplinary approach—combining obstetricians, interventional radiologists, and anesthesiologists—is crucial for optimizing patient safety and fertility outcomes.

#### 4.4.3. Future Directions

Future research should focus on multicenter registries and standardized reporting to enable aggregation of patient-level data. Such efforts would allow refinement of diagnostic criteria, better assessment of prognostic factors, and the development of evidence-based treatment algorithms.

**Figure 3:**
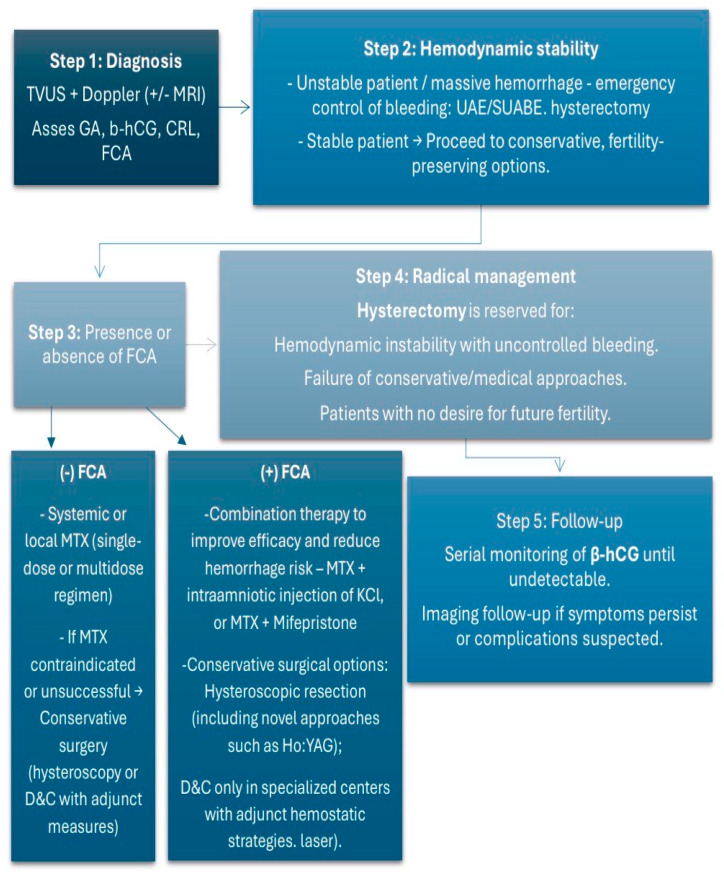
Algorithm for the management of cervical pregnancy.

Based on our findings and comparable data from previously published literature reviews, we developed a proposed algorithm for the management of cervical ectopic pregnancy, presented in Figure 3. This model should be interpreted as an expert opinion–based framework informed by the reviewed evidence rather than a validated clinical guideline. The algorithm emphasizes three key decision points: (1) assessment of hemodynamic stability to determine the need for emergency intervention; (2) evaluation of serum β-hCG levels and fetal cardiac activity to guide the choice between medical and interventional management; and (3) consideration of fertility preservation in selecting the treatment modality. For hemodynamically stable patients with β-hCG < 100,000 mIU/mL and absent cardiac activity, methotrexate-based therapy is recommended as first-line treatment, whereas minimally invasive or surgical approaches (e.g., uterine artery embolization or hysteroscopic evacuation) are reserved for cases with active bleeding or failed medical management. Each decision point reflects patterns repeatedly observed across published cases, emphasizing the central role of clinical stability and reproductive considerations in guiding treatment choice. However, specific thresholds (e.g., β-hCG cut-offs or gestational age limits) could not be standardized due to the limited and heterogeneous data available in the literature. Thus, the algorithm should be viewed as a practical synthesis of observed clinical patterns rather than a validated protocol.

## 5. Conclusions

The assessment of risk factors, early diagnosis, and careful patient selection are crucial for the success of the chosen treatment approach and for improving the overall prognosis. Prompt recognition and individualized management can prevent severe complications, preserve fertility, and optimize patient outcomes.

Conservative approaches should be prioritized as first-line treatment in all cases of cervical pregnancy where fertility preservation is desired. The primary limitation of conservative management is the risk of acute complications, particularly severe hemorrhage. Consequently, nonsurgical treatments should be performed exclusively in specialized medical centers equipped to provide immediate emergency care. As reported in our narrative review, minimally invasive techniques are favored as second-line treatments.

## Figures and Tables

**Table 1:** Overview of cases with cervical pregnancy.

N°	Maternal Age	Parity	Risk Factors	Method of Conception	GA at Diagnosis	Initial β-hCG Levels	Diagnosis	Treatment	Follow-Up
Pascual MA et al. [6]	n.a.	n.a.	n.a.	n.a.	8th g.w.	n.a.	TVUS—cervical twin pregnancy	Intra-amniotic administration of methotrexate under ultrasonographic guidance followed by curettage	Favorable outcome followed by a subsequent intrauterine pregnancy
Astruc A et al. [7]	25	G3P0A2	Induced abortion by D&C, one miscarriage	Spontaneous	9th g.w.	109,850 mIU/mL	TVUS	Uterine artery embolization + in situ methotrexate	β-hCG normalized by day 104; full recovery by 6 months.
Mininni C et al. [8]	43	Nulliparous	n.a.	Spontaneous	9th g.w.	85,220 mIU/mL	TVUS	Methotrexate i.m. + intra-amniotic chloride potassium installation	Rehospitalization due to massive vaginal bleeding 3 months later followed by UAE
Dilday E [9]	45	G3P0A2	LEEP for CIN II	IVF	5 + 1 g.w.	3217 mIU/mL	TVUS	Single dose of intramuscular methotrexate	Nonpregnant levels of β-hCG by day 28
Asante A [10]	n.a.	G2P0	Previous CEP treated with MTX i.m.	Spontaneous	n.a.	n.a.	n.a.	Single dose of intramuscular methotrexate	Uneventful outcome
Sheng [11]	31	G2P0	Left salpingectomy for tubal pregnancy, hysteroscopy for multiple EP	IVF	6 g.w.	119,885 mIU/L	TVUS—heterotopic CEP	US-guided hysteroscopy	Preserving the intrauterine pregnancy, Cesarean section in 38th g.w.
Fan Y [12]—case 1	29	Nulliparous	n.a.	ICSI	6 g.w.	819 mIU/mL	TVUS—heterotopic CEP	Ultrasound-guided aspiration	Preserving the intrauterine pregnancy, Cesarean section in 39 g.w.
Fan Y [12]—case 2	27	G2P1	Right salpingectomy	IVF	6 g.w.	910 mIU/mL 14 days after ET	TVUS—heterotopic CEP	Ultrasound-guided aspiration	Preserving the intrauterine pregnancy, vaginal delivery at 27th g.w. due to PROM
Bolaños-Bravo HH et al. [13]	30	G2P1	Previous Cesarean section	Spontaneous	5 + 4 g.w.	16,189 mIU/mL	TVUS	MTX i.m. 1, 3, 5, 7 day, followed by D&C due to doubled values of β-hCG (35,199 mIU/mL)	Uneventful postoperative period, β-hCG 16 mIU/mL on the 17th day
Terra MEFF et al. [14]	39	G7P0A5	Right tubal pregnancy, treated with MTX, hysteroscopic myomectomy	IVF	10 g.w.	n.a.	TVUS—heterotoic CEP	Spontaneous expulsion of CEP, followed by cervical curettage	Preserving the intrauterine pregnancy, Cesarean section in 39 g.w.
Gun M [15]	39	G2P0A1	D&C of pregnancy at 8th g.w., diathermy of the cervix due to erosion	Spontaneous	6 g.w.	23,060 mIU/mL	TAUS	MTX i.m. on 1 and 7 day	Massive vaginal bleeding on day 11 required surgical intervention—bilateral UAE
Kraemer B et al. [16]	38	G3P2A1	D&C of pregnancy at 9 g.w.	Spontaneous	n.a.	231.4 mIU/mL	Speculum examination—CP on the portio; histological confirmation	Completely excision with biopsy forceps under local anesthesia	Normalization of β-hCG after 7 days
Kouyoumdjian A [17]	22	G2P1A0	Irrelevant	Spontaneous	10 g.w.	n.a.	-	Curettage followed by heavy bleeding and second curettage	Uterine perforation; Total hysterectomy
Oleksik TP et al. [18]—case 1	32	Nulliparous	Irrelevant	Spontaneous	8 g.w.	20,760 mIU/mL	TVUS	MTX i.m. on 1 and 7 day	Increased vascularization detected by Doppler; SUABE followed by decreased vascularization and normalization of β-hCG
Oleksik TP et al. [18]—case 2	31	Nulliparous	Irrelevant	IVF	6 g.w.	13,600 mIU/mL	TVUS	MTX i.m. on 1 and 7 day	Increased vascularization detected by Doppler; SUABE followed by decreased vascularization and normalization of β-hCG
Faschingbauer F et al. [19]	25	G1P0A0	Irrelevant	Conceiving after induction of ovulation with clomiphene citrate	9 g.w.	n.a.	TVUS—heterotopic CEP	Extraction with curettage + Shirodkar cerclage	Uncomplicated postoperative period; Vaginal delivery at 39 g.w.
Tsakos [20]	41	G2P0	CEP after IVF treated with D&C in the past	IVF	7 g.w.	n.a.	TVUS—heterotopic CEP	Aspiration of CEP, followed by cervical Foley catheter placement and Shirodkar cerclage	Uneventful full-term delivery
Moragianni VA et al. [21]	40	G3P1A1	6-week spontaneous miscarriage treated with D&E	IUI	7 + 3 g.w.	n.a.	TVUS	US-guided removal with ring forceps led to heavy vaginal bleeding—US-guided placement of an endocervical Foley catheter	Cesarean section at 39 g.w.
Corticelli A. et al. [22]	34	G2P1	Previous Cesarean section	Spontaneous	5 + 5 g.w.	12,396 mIU/mL	TVUS	MTX i.m. on day 1 and 4, two days later severe vaginal bleeding acquired D&C followed by a Foley ballon tamponade	Uneventful
Mantalenakis, S. et al. [23]	28	Nulliparous	Irrelevant	Spontaneous	12 g.w.	n.a.	TVUS	MTX intraamniotically + i.m. —persistent fetal viability on the 7th day—uneventful curettage	Favorable outcome followed by a subsequent intrauterine pregnancy 5 months later
Davis LB et al. [24]	43	Nulliparous	Irrelevant	IVF	6 g.w.	n.a.	TVUS	Transvaginal ligation of the cervical branches of the uterine artery and injection of vasopressin, followed by D&C	Uneventful
De La Vega GA et al. [25]	35	Nulliparous	Irrelevant	Spontaneous	8 g.w.	n.a.	TVUS	Intracervical infiltration of carboprost, suction curettage of cervix and Foley balloon tamponade	Uneventful
Yıldızhan, B. [26]	43	G2P0A2	Two induced abortions with D&C	Spontaneous	7 g.w.	46,000 mIU/mL	TVUS	Single-dose MTX i.m. + US-guided intra-amniotic administration of MTX	Nonpregnant levels of β-hCG on day 83
Cvetkov D. et al. [27]	32	G1P0	Hysteroscopy for removing a uterine septum	Spontaneous	6 g.w.	5119 mIU/mL	TVUS	Ho:YAG Laser hysteroscopy	Uneventful

**Table 2:** Summary of treatment outcomes in reported cases.

Treatment Modality	Number of Cases (*n*)	Successful (*n*, %)	Failed/Required Additional Intervention (*n*, %)
MTX (local or systemic)	11	5 (45%)	6 (55%)
Uterine artery embolization (UAE/SUABE)	4	4 (100%)	0 (0%)
Dilation and curettage (D&C)	6	4 (67%)	2 (33%)
Hysterectomy	3	3 (100%)	-

**Table 3:** Frequency of reported complications in cervical ectopic pregnancy cases.

Complication	Number of Cases (*n*)	Percentage (% of Total)
Severe vaginal bleeding	4	17%
Hysterectomy	3	12%
Uterine perforation	1	4%
Rehospitalization (delayed complications)	1	4%

**Table 4:** Diagnostic ultrasound criteria for cervical ectopic pregnancy.

Diagnostic Ultrasound Criteria for CEP	
Ultrasound Findings	Additional Doppler Findings
Enlarged cervix with gestational sac inside the cervical canal	Peritrophoblastic blood flow around the gestational sac detected with color Doppler
Absence of gestational sac inside the uterine cavity	
Enlarged uterus	
Diffuse irregular echoes within the uterus	
Absence of sliding sign	

## Data Availability

Data presented in this study are available on request from the corresponding author due to privacy concerns.

## Supplementary Materials

The following supporting information can be downloaded at: https://www.mdpi.com/article/10.3390/medicina61112072/s1, Table S1: Completeness of data reporting across included case reports of cervical ectopic pregnancy.

## Abbreviations

n.a.	not available
CEP	cervical ectopic pregnancy
GA	gestational age
g.w.	gestational weeks
TVUS	transvaginal ultrasound
TAUS	transabdominal ultrasound
UAE	Angiographic uterine artery embolization
EP	endometrial polyps
ET	embryo transfer
PROM	premature rupture of membranes
SUABE	super-selective embolization of the pathological uterine arteries branch
IUI	intrauterine insemination
CRL	crown-rump length
FCA	fetal cardiac activity
