# Supplementary material for: Current Trends in the Treatment of Cervical Pregnancy: A Narrative Review
Source: Medicina (Kaunas). 2025 Nov 20;61(11):2072. doi: 10.3390/medicina61112072 (PMC12654775; doi:10.3390/medicina61112072)
Supplement: Supplementary file 1 [file medicina-61-02072-s001.zip › medicina-3914936-supplementary.pdf]

Supplementary Table S1. Completeness of data reporting across included case reports of cervical ectopic pregnancy.

(+ = data reported; – = data not reported)

№	Maternal Age	Parity	Risk factors	Method of conception	GA at diagnosis	Initial β-hCG levels	Diagnosis	Treatment	Follow-up
Pascual MA et al. [6]	-	-	-	-	+	-	+	+	+
Astruc A et al. [7]	+	+	+	+	+	+	+	+	+
Mininni C et al. [8]	+	+	-	+	+	+	+	+	+
Dilday E [9]	+	+	+	+	+	+	+	+	+
Asante A [10]	-	+	+	+	-	-	-	+	+
Sheng [11]	+	+	+	+	+	+	+	+	+
Fan Y [12] – case 1	+	+	-	+	+	+	+	+	+
Fan Y [12] – case 2	+	+	+	+	+	+	+	+	+
Bolaños-Bravo HH et al. [13]	+	+	+	+	+	+	+	+	+
Terra MEFF et al. [14]	+	+	+	+	+	-	+	+	+
Gun M [15]	+	+	+	+	+	+	+	+	+
Kraemer B et al. [16]	+	+	+	+	-	+	+	+	+
Kouyoumdjian A [17]	+	+	+	+	+	-	-	+	+
Oleksik TP et al. [18] – case 1	+	+	+	+	+	+	+	+	+
Oleksik TP et al. [18] – case 2	+	+	+	+	+	+	+	+	+
Faschingbauer F et al. [19]	+	+	+	+	+	-	+	+	+
Tsakos [20]	+	+	+	+	+	-	+	+	+
Moragianni VA et al. [21]	+	+	+	+	+	-	+	+	+
Corticelli A. et al [22]	+	+	+	+	+	+	+	+	+
Mantalenakis, S. et al [23]	+	+	+	+	+	-	+	+	+
Davis LB et al. [24]	+	+	+	+	+	-	+	+	+
De La Vega GA et al. [25]	+	+	+	+	+	-	+	+	+
Yıldızhan, B. [26]	+	+	+	+	+	+	+	+	+
Cvetkov D. et al [27]	+	+	+	+	+	+	+	+	+
